# The overlooked first intercostal ligament: Does it help to stabilize the Weberian apparatus?

**DOI:** 10.1111/joa.14168

**Published:** 2024-11-18

**Authors:** Jake Leyhr, Tatjana Haitina, Nathan C. Bird

**Affiliations:** ^1^ Department of Organismal Biology Uppsala University Uppsala Sweden; ^2^ Department of Biology University of Northern Iowa Cedar Falls Iowa USA

**Keywords:** 3D segmentation, histology, iodine staining, propagation phase‐contrast synchrotron microtomography, zebrafish

## Abstract

The Weberian apparatus is a novel hearing adaptation that facilitates increased hearing sensitivity in otophysan fishes. The apparatus is a complex system composed of modifications to anterior vertebral elements, the inner ear, and the swim bladder. A critical piece of the system that often receives minor attention are the various ligaments that bridge these three regions. The most famous of the ligaments is the interossicular ligament, which connects the Weberian ossicle chain (scaphium–intercalarium–tripus). Several other ligaments are present, including the suspensor (tripus to parapophysis 4) and the triple ligament (tripus–os suspensorium–tunica externa). Here, by combining diffusible iodine‐based contrast enhancement (DICE) and propagation phase‐contrast synchrotron radiation micro‐computed tomography (PPC‐SRμCT) with classic histological methods, we shine new light on the first intercostal ligament (ICL1) and discuss its potential function in relation to the Weberian apparatus. ICL1 is nearly absent from the cypriniform literature, typically only mentioned in a general discussion together with other intercostal ligaments. This study examines the development and structure of ICL1 comparatively with the other definitive Weberian ligaments in the zebrafish (*Danio rerio*). We provide a comprehensive view of three‐dimensional shape, development, and composition to generate hypotheses regarding potential functions of ICL1 within the greater Weberian apparatus. Given new detail presented herein regarding the structure of ICL1, modifications to rib 5 and parapophysis 4 for ICL1 attachment, and the alignment of ICL1 with the os suspensorium, we propose a supportive (anchoring) role of ICL1 to aid in minimizing non‐optimal movement of the structures of the fourth vertebra. This addition would focus vibrations anteriorly through the ossicle chain with minimal signal loss in zebrafish and other species with similar Weberian apparatus morphologies. We conclude that ICL1 should be included in future analyses of Weberian apparatus function where ligaments are addressed.

## Graphical abstract

3D rending of adult zebrafish Weberian apparatus in association with the occiput, swim bladder, and rib‐bearing vertebrae, with highlighted interossicular, suspensor, and intercostal ligaments.

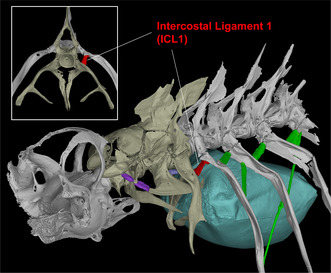

## 1 INTRODUCTION

The Weberian apparatus is a complex and novel morphological system used to enhance hearing in a diverse collection of fishes and unites the more than 10,000 species in Series Otophysi, the largest group of primarily freshwater fishes (Nelson et al., 2016). The bony and soft tissue components of the Weberian apparatus vary substantially among species, both phylogenetically among the orders (Cypriniformes, Characiformes, Siluriformes, and Gymnotiformes), as well as within orders in various environmental systems, such as within Cypriniformes (Alexander, 1962; Bird, Abels, & Richardson, 2020; Bird & Hernandez, 2007) and Siluriformes (reviewed in Ladich, 2023). The morphological variation is likely intimately tied to unique noise regimes created in these different environments, and various modifications in both hard and soft tissues (Bird, Abels, & Richardson, 2020) are employed to maintain optimal functionality. This is evidenced by compromised functionality when shifting to different environments (Amoser & Ladich, 2005); however, some plasticity has been found among cypriniforms in different environments (Ladich, 1999).

**FIGURE 1:**
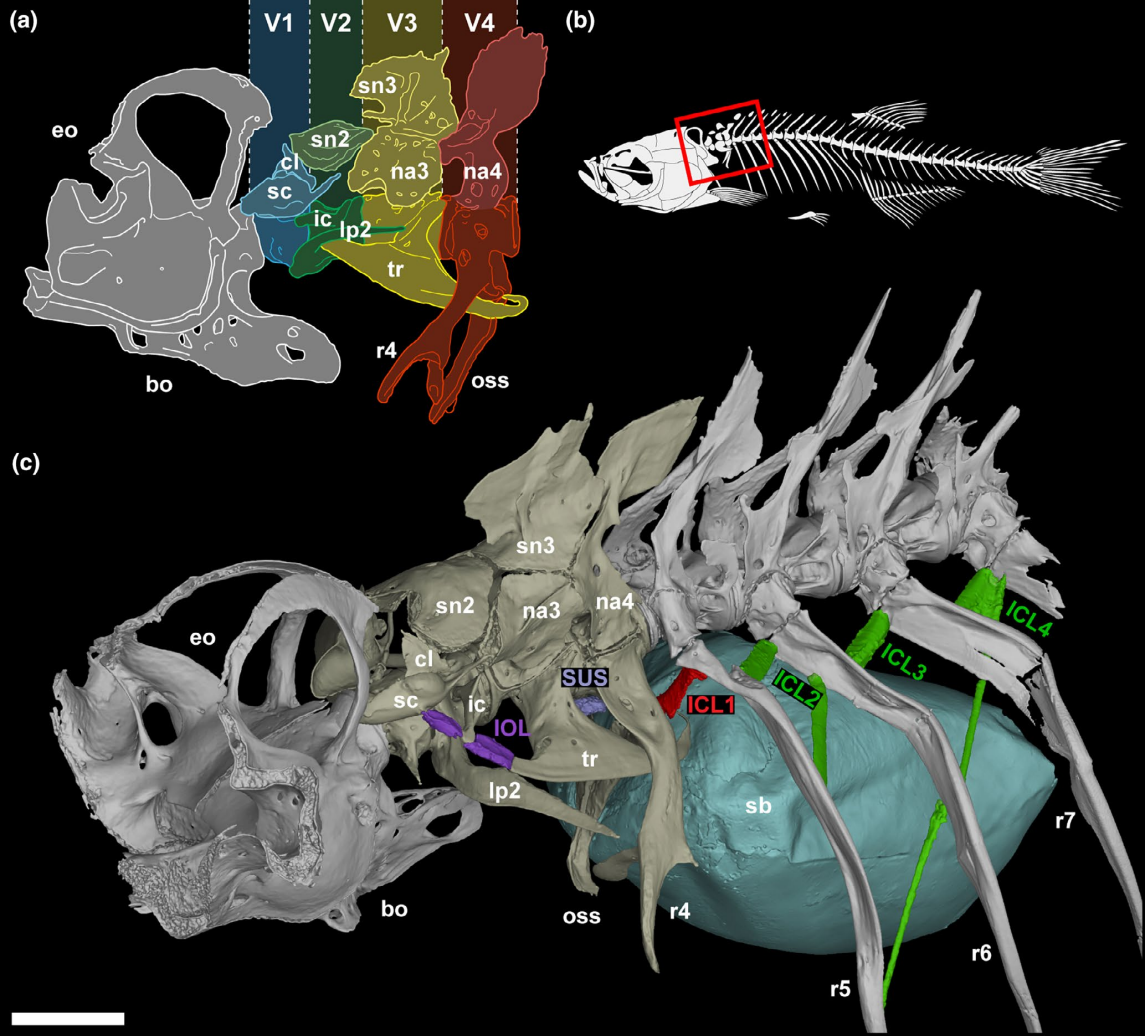
(a) Schematic of the adult zebrafish Weberian apparatus (cervical vertebrae 1–4) and occiput. (b) Schematic of zebrafish skeleton with red box highlighting the region containing the occiput, Weberian apparatus, and anterior rib‐bearing vertebrae displayed in (c). (c) 3D rending of 24 mm SL zebrafish Weberian apparatus in association with the occiput, swim bladder, and rib‐bearing vertebrae, with interossicular, suspensor, and intercostal ligaments highlighted in purple, lilac, red, and green. Ventrally projecting bones from the right‐hand side of the vertebrae were removed to simplify the visualization. bo, basioccipital; cl, claustrum; eo, exoccipital; ic, intercalarium; ICL, intercostal ligament; IOL, interossicular ligament; lp, lateral process; na, neural arch; oss, os suspensorium; r, rib; sb, swim bladder; sc, scaphium; sn, supraneural; SUS, suspensor ligament; tr, tripus; V, vertebra. Scale bar = 500 μm.

The Weberian apparatus consists of several modifications to the inner ear, swim bladder, and anterior vertebrae compared to non‐otophysans. The most famous elements are the Weberian ossicles (claustrum, scaphium, intercalarium, tripus, and os suspensorium), and are easily identified and examined in the relatively simple Weberian apparatus morphology seen in most cyprinids, such as the zebrafish (Bird & Mabee, 2003). The scaphium, intercalarium, and tripus act as a chain for direct transmission of vibrations generated by the swim bladder (in response to sound) to the sinus impar (then into the ear) via ligamentous attachments (Figure 1). The vertebral elements of the Weberian apparatus are modifications of several skeletal elements, including neural arches (scaphium, intercalarium) and parapophyses and ribs (tripus, os suspensorium). While the claustrum and os suspensorium are not generally attributed a primary role in Weberian apparatus function, their intimate association with the ossicle chain and the sinus impar (claustrum) and swim bladder (os suspensorium) suggests a supportive or stabilization role, rather than transmissive. Collectively, the apparatus transforms pressure waves collected by the swim bladder and transmits (and likely amplifies) them to the inner ear where they can be detected by differential motion in the otolith‐based inner ear (Ladich & Popper, 2004; Ladich & Schulz‐Mirbach, 2016). This allows for detection of wider frequency ranges as well as sensitivity at lower sound pressures compared to species with non‐modified hearing (Fay, 1988; Fay & Simmons, 1999; Schellart & Popper, 1992).

### 1.1 Weberian apparatus studies in cypriniform fishes

A rich lineage of descriptive research is found for the Weberian apparatus, especially for catfishes, dating back to Weber's initial description of *Silurus glanis* (1819, 1820; see Ladich, 2023 for historical review). The research history for cypriniform fishes is nearly as long, and work on evolutionary variation, development, function, and genetics is available for both groups. For simplicity, we will focus primarily on the work done with cypriniform fishes. Substantial morphological research has focused on the bony ossicles and other skeletal elements, including various bony encapsulations of the swim bladder (Bird, Abels, & Richardson, 2020; Ladich, 2023). Limited research has examined the structure, development, and evolution of the various ligamentous components within the vertebral region. The ligaments in this region play a pivotal role in proper transmission of motion between the ossicles by physically linking them together. Several ligaments are of note, the most commonly referenced being the interossicular ligament. However, other ligaments are present and vary substantially in fiber density and cellularity, suggesting a variety of functional roles within the system (Bird, Abels, & Richardson, 2020).

Few comprehensive ontogenetic studies of the cypriniform Weberian apparatus are available. However, detailed studies on ontogeny in the zebrafish can be found for each region of the Weberian apparatus, including the inner ear (Bang et al., 2001; Bever & Fekete, 2002; Haddon & Lewis, 1996; Kimmel et al., 1995; Wang et al., 2015), vertebrae and ossicles (Bird & Mabee, 2003; Grande & Young, 2004), and swim bladder (Finney et al., 2006; Robertson et al., 2007; Winata et al., 2009). An ontogenetic study of the complete Weberian apparatus is also available for zebrafish (Bird, Abels, & Richardson, 2020).

### 1.2 Review of ligaments and previous ligament research

Within the Weberian apparatus, four confirmed ligaments have been discussed within the literature to various degrees of depth: Interossicular (anterior and posterior; herein “Interossicular” refers to both segments unless specified), Suspensor, and Triple ligaments. We propose a fifth ligament, Intercostal 1 (between Rib 4 and Rib 5), merits additional attention based on its location, attachment, composition, and the likely role of intercostal ligaments in the evolution of the Weberian apparatus. Each of these ligaments are reviewed below.

#### 1.2.1 Interossicular

The most obvious and critical ligament in the system is the interossicular ligament (IOL), which ties the anterior process of the tripus to the manubrium of the intercalarium (IOL posterior), and the manubrium to the lateral surface of the scaphium (IOL anterior). The interossicular ligament, therefore, plays a direct role in sound transmission in the apparatus, is a key component for functionality, and is typically described or mentioned in most species‐level descriptions of the Weberian apparatus. The interossicular ligament has a particular histological signature, being fiber dense and likely high tensile strength.

While nearly uniformly referred to as a ligament, Chranilov (1927) referred to the IOL as a tendon. The IOL has dense collagen fibers identified by multiple stains, including Hall Brundt's Quadruple Stain (Bird, Abels, & Richardson, 2020; Bird, Richardson, & Abels, 2020) and Mallory's triple stain (Bird & Mabee, 2003). Alexander (1962) also noted that the IOL contains ichthyocol (most often associated with the swim bladder), which is collagen arranged morphologically to appear like short needles. While likely a form of collagen I, the relationship of ichthyocol to other collagens has not been analyzed molecularly.

#### 1.2.2 Suspensor ligament

Another large ligament is the suspensor ligament, which attaches the base of the tripus to the base of parapophysis 4 (which bears rib 4 and the os suspensorium). This ligament tends to be much less fiber dense (Alexander, 1962) and much higher in cellularity (Bird, Richardson, & Abels, 2020; named the tripus–parapophysis 4 ligament). Based on chemical staining, Alexander (1962) described this ligament as composed of elastin, with collagen absent, making it starkly different from the IOL; however, no molecular analyses are available that could support this. While its specific role is unclear, it likely plays a supportive role in limiting the rocking of these elements in specific planes, possibly to help minimize signal loss during sound transmission.

Several studies have mentioned the suspensor ligament with various levels of description. Nelson (1948) described the suspensor in Catostomidae (however, he did not explicitly name it) and postulated that it kept the tripus in place along with the triple ligament. Chardon and Vandewalle (1997) mentioned a “suspensor” ligament; however, based on location, connectivity, shape (thick and crescent‐shaped), and composition (collagen), they likely described the triple ligament. The suspensor ligament has also been described in *Gobio* (Vandewalle, 1974), *Barbus* (Vandewalle et al., 1989), zebrafish (Bird, Richardson, & Abels, 2020; Leyhr, Sanchez, et al., 2023), *Botia* (Alexander, 1964a), and more broadly across cypriniforms (Alexander, 1962; Bird, Abels, & Richardson, 2020).

#### 1.2.3 Triple ligament

The triple ligament is a small ligament that connects the transformator process of the tripus, os suspensorium, and swim bladder (Bird, Abels, & Richardson, 2020; Bird, Richardson, & Abels, 2020). Some early descriptions in cyprinids (Evans, 1925) and *Hybognathus* (Niazi & Moore, 1962) mistook this ligament for a muscle, hence the alternate name of “tensor tympani” in early literature, and may have led to several instances of the ligament being described but unnamed in the mid‐1900s. Nelson (1948) described it (unnamed) as having a role in maintaining the position of the tripus. Mookerjee et al. (1952) described it (also unnamed) as a band of connective tissue connecting the os suspensorium and the transformator process in *Esomus*. Rojo (1987) also described the ligament in *Barbus* but left it unnamed.

The triple ligament in *Danio* was described by Bird, Richardson, and Abels (2020) as a dense, collagen‐rich ligament, roughly fan‐shaped. Bird, Abels, and Richardson (2020) also described it in *Danio*, *Gyrinocheilus*, and *Ambastia*, but left it unnamed. A similar description was found by Alexander (1962; Ligament 1), who described it as fan‐shaped bundles of radiating collagen fibers. Chardon and Vandewalle (1997) postulated that the triple ligament was a specialized intercostal ligament under the assumption that the os suspensorium is a modified Rib 4 while the transformator process is a modified Rib 3.

#### 1.2.4 Intercostals

Intercostal ligaments are most often omitted from descriptions of the Weberian apparatus. When present, they are typically drawn in figures but not described in the text. A variety of intercostal ligament patterns and lengths are found in many species, including cyprinids (Liao & Kullander, 2013). The first intercostal ligament is found bridging and uniting the head of rib 5 to the region where rib 4 and the os suspensorium split on parapophysis 4, based on position and connectivity this ligament could provide specific support to the structures of vertebra 4, limiting their movement (likely restricting dorsal–ventral flexion or rotation at the centrum). The intimate association of parapophysis/rib 4 and parapophysis/rib 5 has been noted in other cypriniform species (Conway, 2011).

Many questions still remain regarding the origin and homologies of both the skeletal elements of the Weberian apparatus and its ligaments. Rosen and Greenwood (1970) hypothesized that the interossicular ligament was likely homologous to ancestral inter‐neural arch ligaments (IOL anterior, scaphium to intercalarium) and a combination of inter‐neural arch and neural arch–parapophysis ligaments (IOL posterior; intercalarium to tripus). This would make sense given the consensus homologies of the scaphium (neural arch 1), intercalarium (neural arch 2), and tripus (largely parapophysis 3); however, questions remain regarding the ubiquity of these types of ligaments in most fishes. Unfortunately, they did not address the suspensor or triple ligaments, but based on their drawings one could make a strong claim that their intercostal ligament between R3 and R4 would be the precursor to the suspensor ligament. Intercostal ligaments were explicitly drawn by Chardon and Vandewalle (1997), including between R4 and R5. Following the initial suggestion of Sagemehl (1885), they hypothesize that the IOL represents the transformation of a swim bladder outgrowth, similar to those found in clupeids, into an elongated ligament. Their hypothetical ancestral reconstruction has a broad, dense collagenous ligament extending between the tripus and parapophysis/rib 4, suggesting an intercostal origin for at least the suspensor ligament, in agreement with Alexander (1962).

For this study, we reexamine this ligament and compare the structure and development of the first intercostal ligament with the other main Weberian ligaments to generate hypotheses regarding its possible functional role within the system. Similar histological signatures, specific insertion sites, and developmental timing may provide evidence of the importance of this ligament, as well as a broader role of vertebra 5 in the simplified zebrafish Weberian system (generally considered limited to V1 through V4). Taken with other morphological elements of vertebra 5, a reexamination of this vertebra in the sphere of Weberian apparatus evolution is warranted, as it may represent a transitional vertebra that develops, at least in part, within the Weberian apparatus developmental module and play a functional role within the Weberian system.

## 2 MATERIALS AND METHODS

### 2.1 DICE‐PPC‐SRμCT

Two juvenile zebrafish (*Danio rerio*, AB line) at 1 and 2 months postfertilization, 8.5 mm and 24.0 mm standard length (SL), respectively, were euthanized with an overdose of MS‐222 (300 mg/L) and fixed in 4% paraformaldehyde. Diffusible iodine‐based contrast enhancement and propagation phase‐contrast synchrotron radiation micro‐computed tomography (DICE‐PPC‐SRμCT) was performed as previously described by Leyhr et al. (Leyhr, Sanchez, et al., 2023) and images obtained at BM05 of the European Synchrotron Radiation Facility–Extremely Brilliant Source (ESRF‐EBS) in France with voxel sizes of 0.727 and 3 μm. Reconstructed jpeg2000 image stacks were imported into VGStudio MAX (v3.5.1) for manual segmentation and rendering.

All animal experimental procedures were approved by the local ethics committee for animal research in Uppsala, Sweden (permit number 5.8.18‐18,096/2019). All procedures for the experiments were performed in accordance with the animal welfare guidelines of the Swedish National Board for Laboratory Animals.

### 2.2 Histology

Juvenile and adult zebrafish *Danio rerio*, AB line, 5.5–33.7 mm total length (TL) were obtained from the Zebrafish International Resource Center (Eugene, Oregon). Adults were anesthetized using buffered 0.04% MS‐222, fixed in chilled 10% buffered formalin (24 h at 4°C minimum). Three specimens were paraffin‐embedded and sectioned at 8 mm (one specimen per plane—sagittal, horizontal, and coronal) and processed as previously described (Bird, Abels, & Richardson, 2020). Sections were stained using a modified Hall–Brunt Quadruple stain (Hall, 1986), allowing for visualization of ligaments as well as bone, cartilage, and ossification sites within cartilages. Bone and collagen I‐rich ligaments stain red, elastin fibers stain blue/gray, and cartilage stains blue.

Histological images were collected using a Zeiss Axio ScopeA.1 microscope with a ProgRes CF Scan camera and CapturePro v2.8.8 software (Jenoptik). When necessary, composite histological images were assembled in Adobe Photoshop 2022 using the Automate‐Photomerge feature (default settings) to remove stitching errors.

All histological‐based procedures followed an approved University of Northern Iowa IACUC protocol (#2015‐05‐1). All tissues are deposited in Biological Collections in the University of Northern Iowa Department of Biology.

## 3 RESULTS

### 3.1 Structural comparison of intercostal ligament 1 to Weberian ligaments and posterior intercostal ligaments

**FIGURE 2:**
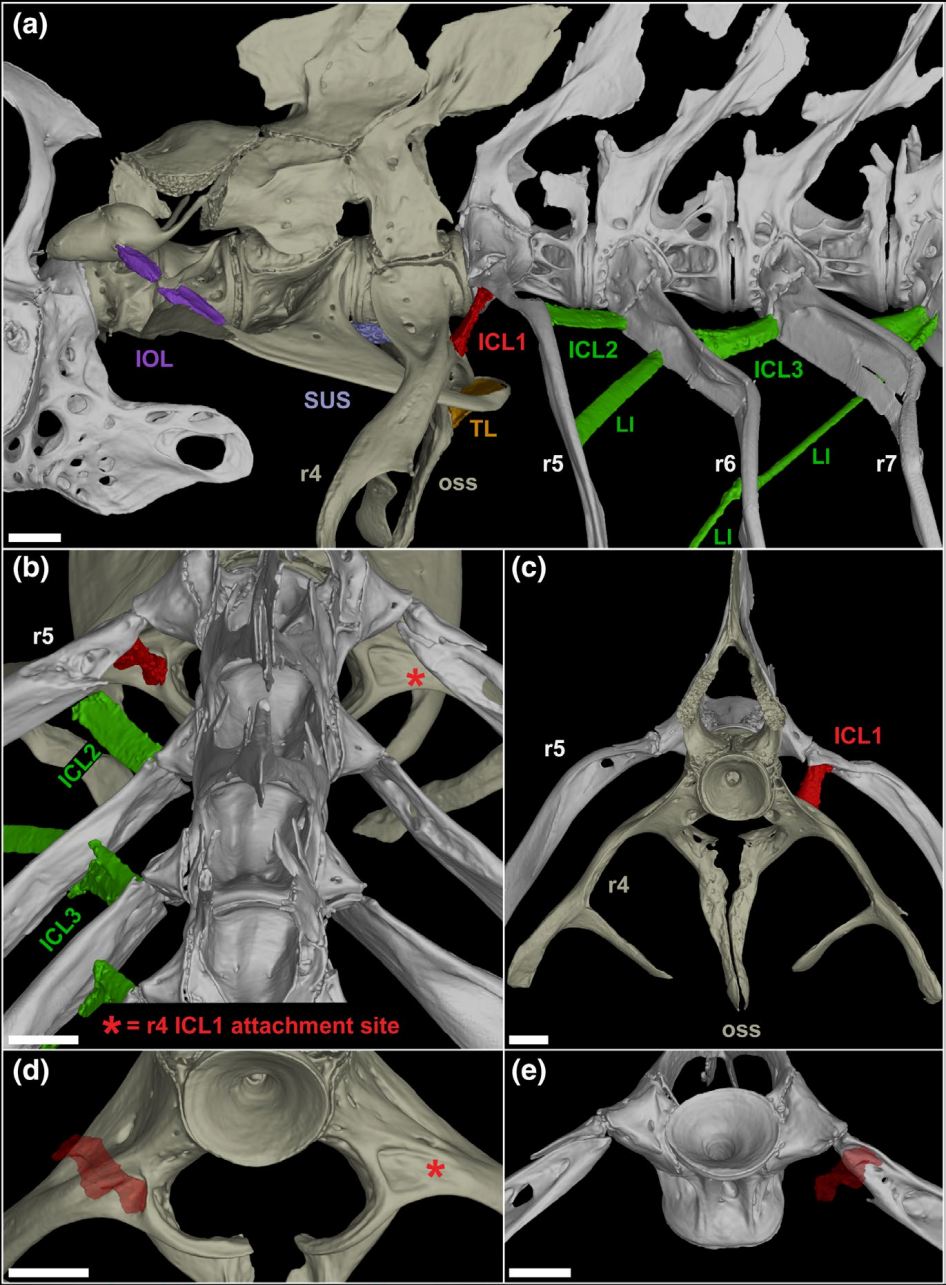
3D renderings of the intercostal ligaments of a 24 mm SL zebrafish. (a) lateral view of vertebrae 1–7 including the Weberian and intercostal ligaments. (b) posterodorsal view of vertebrae 5–7 with only the left intercostal ligaments rendered. (c) anterior view of vertebrae 4 and 5 with the left ICL1 rendered. (d) posterodorsal view of the ICL1 attachment site (red asterisk) on rib/parapophysis 4. (e) anteroventral view of the ICL1 attachment site on rib 5. ICL, intercostal ligament; IOL, interossicular ligament; LI, long intercostal; oss, os suspensorium; r, rib; SUS, suspensor ligament; TL, triple ligament. Scale bar = 200 μm.

The first intercostal ligament (ICL1), connecting rib 5 to parapophysis (rib) 4 has similarities to both Weberian ligaments and more posterior intercostal ligaments, as well as unique features bridging the different sets of ligaments (Figure 1c). ICL1 shows distinct differences with the more posterior ICLs, indicating a potential for slightly different functions. The posterior ICLs (see Figure 2 for ICL2 and ICL3) appear to have more slack compared to ICL1. All ICLs have an origin near the proximal end of the rib head of the more posterior rib, and attach more distally (proximal quarter) to the next anteriormost rib, typically along a flange or ridge in the adult rib. ICL1 differs in that it connects medially, rather than laterally, and at a steep vertical angle (Figure 2) in a posterior depression marking the splitting point of rib 4 and the os suspensorium. The posterior ICLs are dorsal‐ventrally flattened and oriented primarily in the anterior–posterior plane (projecting antero‐laterally, Figure 2b), while ICL1 is more rounded, varies in width along its length (resembling an hour‐glass shape), and is oriented in the dorsal–ventral plane (projecting ventro‐medially, Figure 2b–e). This shift in direction aligns it with the os suspensorium, suggesting potential activation upon os suspensorium motion.

**FIGURE 3:**
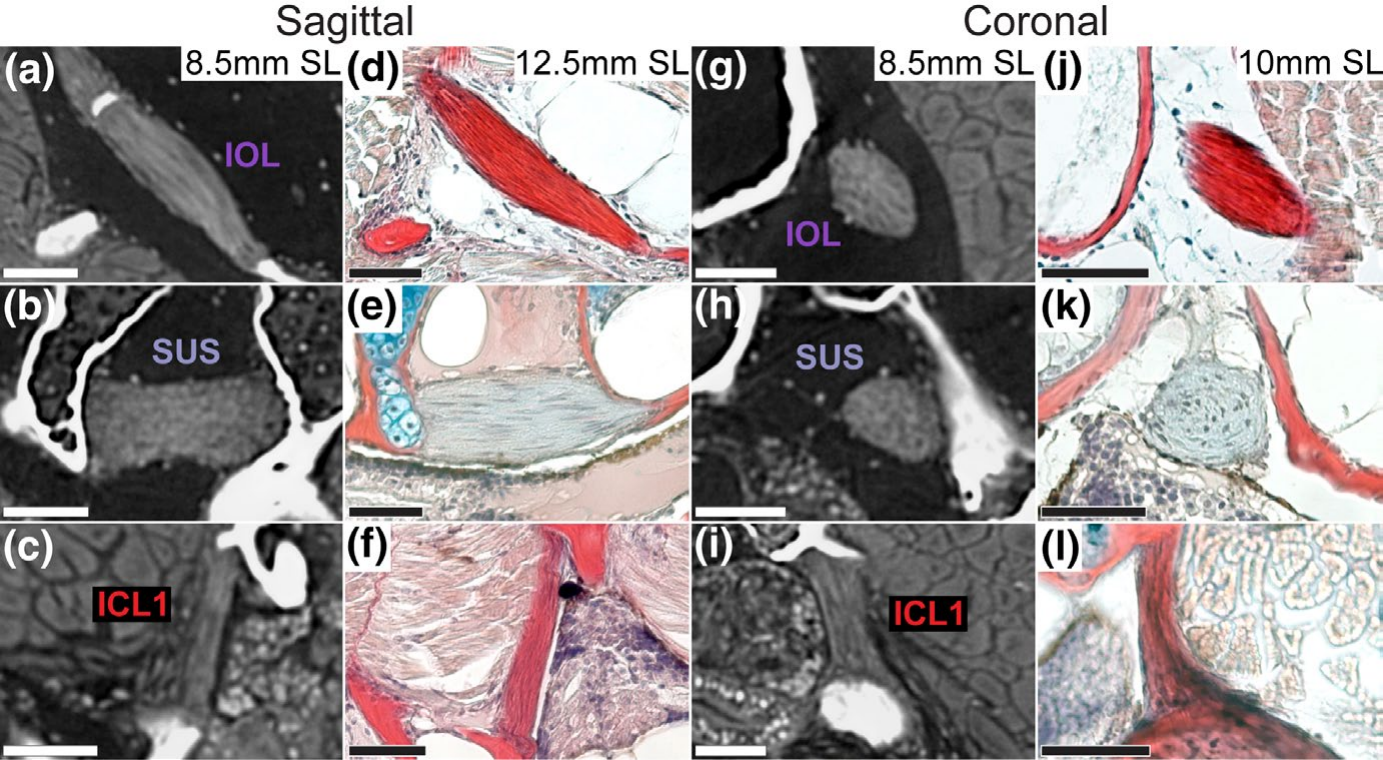
Comparison of sagittal (a–f) and coronal (g–l) virtual and histological thin sections through the Weberian ligaments and ICL1 of zebrafish. ICL, intercostal ligament; IOL, interossicular ligament; SUS, suspensor ligament. Scale bar = 40 μm.

**FIGURE 4:**
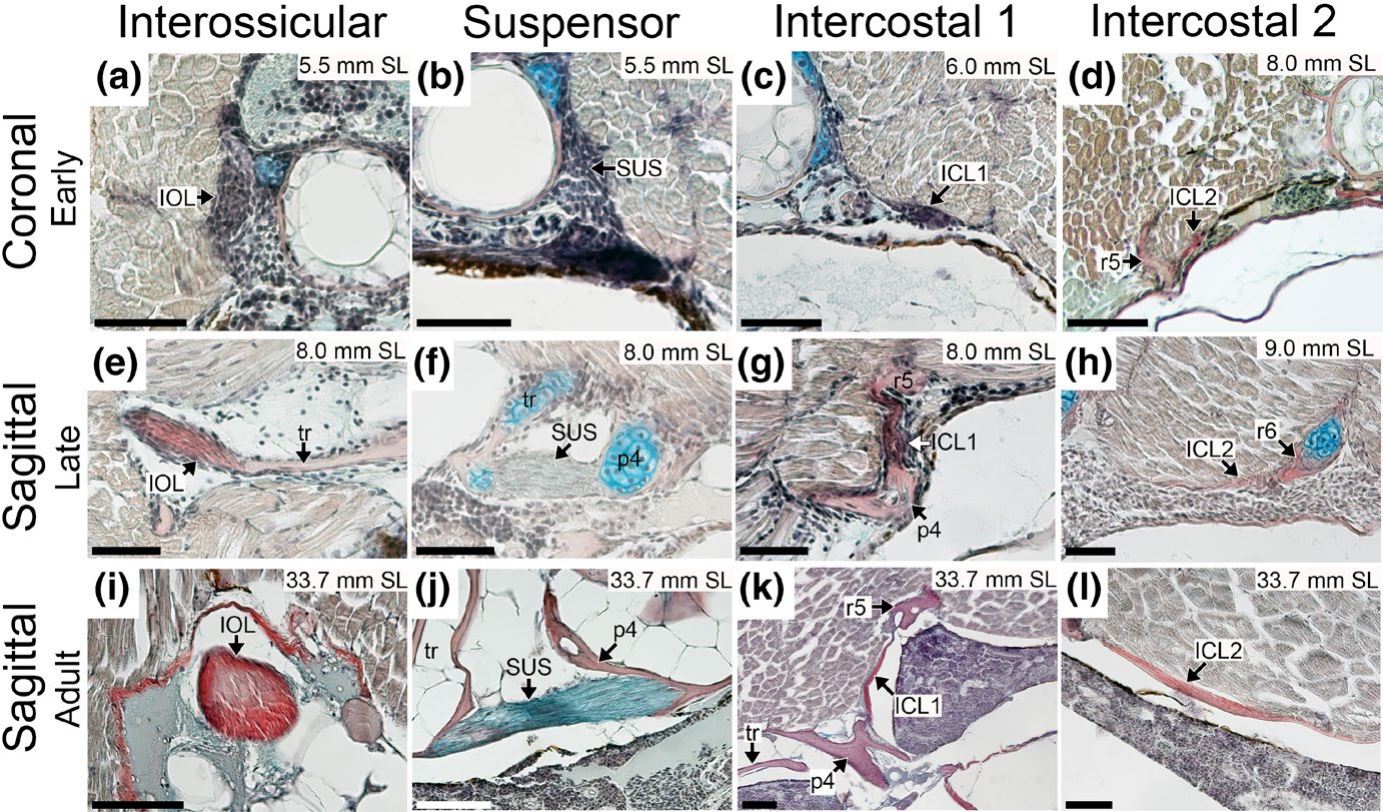
Comparative development of Weberian and intercostal ligaments in zebrafish. (a–d) in coronal plane, (e–l) in sagittal plane (anterior to the left and dorsal to the top in all sagittal images). ICL, intercostal ligament; IOL, interossicular ligament; p4, parapophysis 4; r, rib; SUS, suspensor ligament; tr, tripus. Scale bar = 40 μm.

**FIGURE 5:**
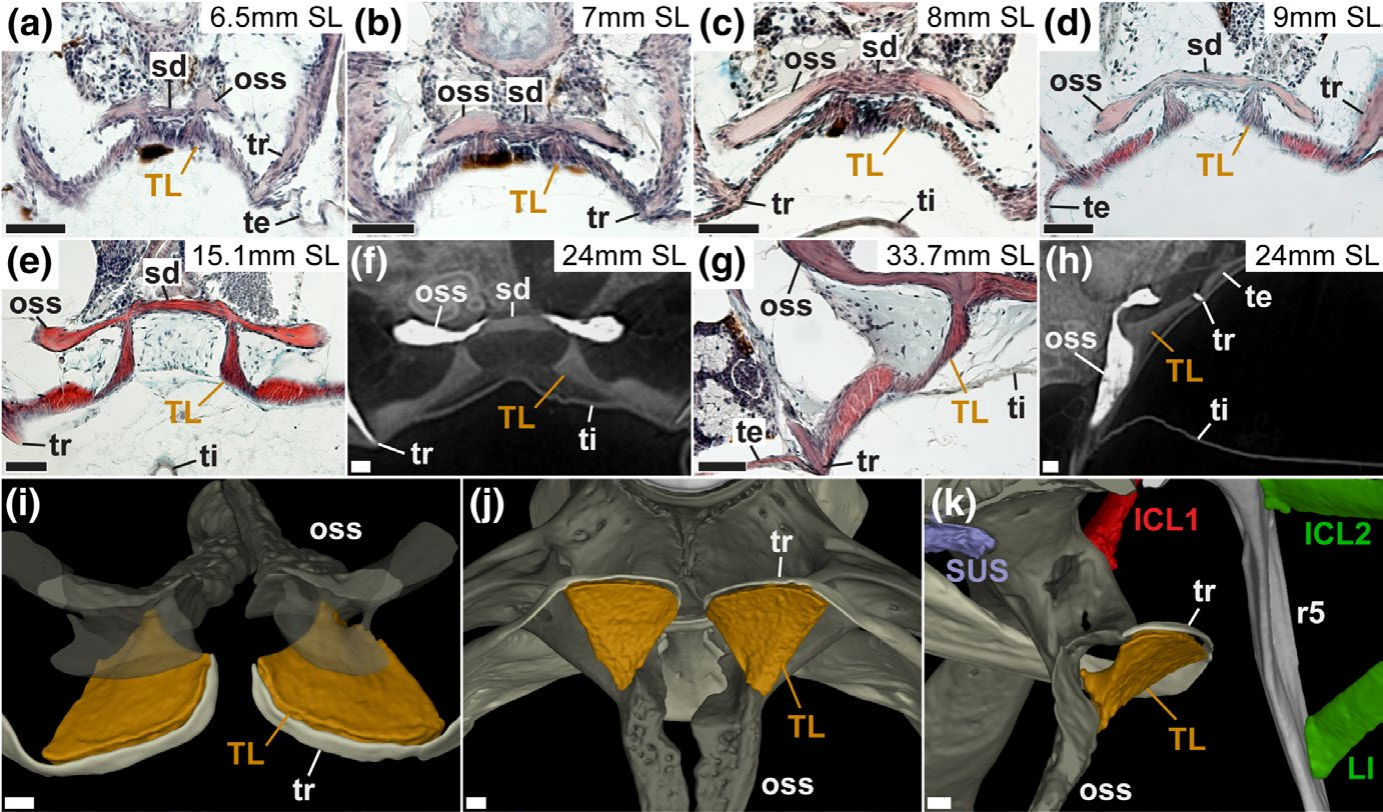
Comparative development (a–h) and reconstructed adult morphology (i–k) of the triple ligament. (a–g) in horizontal plane (anterior to the top), (h) in sagittal plane (anterior to the left and dorsal to the top), (i) anterodorsal view of the triple ligament with partially transparent os suspensorium, posteroventral (j) and medial (k) view of the triple ligament. ICL, intercostal ligament (number denotes order starting from rib 4 to rib 5); LI, long intercostal; oss, os suspensorium; r, rib (number denotes vertebral level); sd, syndesmosis; SUS, suspensor ligament; te, tunica externa; ti, tunica interna; TL, triple ligament; tr, transformator process of the tripus. Scale bar = 40 μm.

The Weberian ligaments vary the most in composition, with the suspensor ligament (SUS, Figure 3, middle row) having a markedly different histological signature (elastin: blue/gray) compared to the strong collagen 1 staining (red) seen in the interossicular ligament (IOL, Figure 3, top row). ICL1 is remarkably similar to the IOL (Figure 3, bottom row), ICL2 (Figure 4), and the triple ligament (Figure 5), being composed of densely packed collagen‐1‐positive fibers. ICL1 and the IOL segments also share a taut fiber arrangement compared to the SUS and the posterior ICLs. ICL1 also has a defined ovoid shape, especially in its attachment regions, which is similar to the Weberian ligaments rather than the other intercostals, which are more flattened in appearance.

### 3.2 Ontogenetic development of Weberian ligaments

The first indication of ligamentous development is at 5.5 mm SL, with mesenchymal condensations (fibroblasts) near the condensing cartilage elements in the relative positions of the interossicular ligaments and the suspensor ligament, but not the triple ligament or intercostals (Figure 4a,b). At 6.0 mm SL, definitive staining of collagen fibers is present for the interossicular ligaments as well as diffuse connective tissue staining for the suspensor ligament (Data not shown; Bird, Richardson, & Abels, 2020). At this stage, fibroblasts are seen in a large condensation in the location of the first intercostal ligament (Figure 4c). No clear development was seen for more posterior intercostal ligaments; however, loose aggregations of cells can be seen. No development of the triple ligament was noted.

Development of the ligaments continues rapidly, with fiber deposition in ICL1 beginning at 6.5 mm SL, as well as continued growth of the interossicular and suspensor ligaments, which are composed of more fibroblasts than fibers. A small condensation is found in the presumptive location of the triple ligament (Figure 5a). By 7.0 mm SL, ICL1 is increasing in size and fiber content, while clear initial fiber staining can be seen for the triple ligament (Figure 5b) and posterior intercostal ligaments, as well as initial condensations for the more posterior intercostal ligaments. The IOLs and suspensor ligament continue to increase in fiber content, now reaching approximately a 50–50 split in cells versus fibers in their composition (Data not shown; Bird, Richardson, & Abels, 2020).

By 8 mm SL, both ICL1 and the suspensor ligament continue growing (Figure 4f,g), ICL1 now with substantial cellularity. The IOL ligaments have transitioned now to a majority of fibers rather than fibroblasts, now densely packed with collagen‐positive fibers (Figure 4e). Both the posterior ICLs (Figure 4d) and the triple ligament (Figure 5c) have become more distinct, but significantly smaller and less fiber dense compared to the more anterior ligaments. By 9 mm SL, all ligaments continue getting larger, with ICL1 and the IOLs increasing in fiber clarity. Definitive posterior ICLs are now clearly identified and gaining fiber density (Figure 4h). The suspensor ligament maintains a high level of cellularity, and its fibers are not as distinct, due to the difference in composition. Both the triple ligament (Figure 5d) and posterior intercostals have clearly defined fibers (less robust than anterior ligaments), and have taken more of an adult shape.

By 10 mm SL, all ligaments have reached near adult morphology, with shape, composition, fiber density, and cellularity approximating the adult form. Between 10 and 15 mm SL, no significant changes were seen in the ligaments, rather growth and size change relative to overall body growth, which continued through adult stages (Figures 4i–l and 5e–k).

## 4 DISCUSSION

### 4.1 Intercostal ligament 1 as a Weberian ligament, and vertebra 5 as a transitional vertebra

ICL1 shares similarities with the IOL, and marked differences from other ICLs suggest that it should be included within the group of Weberian ligaments. The most critical evidence lies in its attachment to vertebra 4. The taut, tubular ICL1 originates from a socket in rib 5, and inserts in a groove/depression in parapophysis 4, an arrangement not seen in any other ICL. The switch from anterior–posterior orientation to dorsal–ventral likely allows for parapophysis 4 (and its distal projections) to be strongly anchored to rib 5, potentially minimizing non‐optimal motion of the structure that would otherwise reduce Weberian apparatus sensitivity due to unfocused os suspensorium motion.

ICL1 is not alone in its differences with posterior elements, vertebra 5 also varies significantly from posterior rib‐bearing vertebrae. Parapophysis 5 is substantially more dorsally positioned compared to those of more posterior vertebrae. Neural arch 5 is often taller (more dorsal height) and narrower than posterior neural arches. Importantly, rib 5 varies substantially compared to other ribs, having a shorter and more rounded head (to match the parapophysis articulation site), and is more slender along its entire length (other ribs tend to have broad, flat plates in their proximal quarter).

In a comparative perspective, the concept of vertebra 5 as a Weberian vertebra is not novel or surprising. Particularly in many catfish species (Siluriformes), the incorporation of vertebra 5 and its structures into the Weberian apparatus structure is well established. In the developing structure of *Clarias gariepinus*, the basiventrals (parapophyses) of vertebra 5 are incorporated into the broader Weberian structure (Radermaker et al., 1989). Incorporation of the fifth parapophysis into a thin sheet (plate) overlying the swim bladder to the skin, or expanded to a near capsule, was noted in primitive Siluriforms (including *Clarias*) by Alexander (1964b). These species, as well as *Plecostomus*, often incorporate centrum 5 into their fused *complex centrum* (Alexander, 1964b). Chardon et al. (2003) also described various contributions of elements of the fifth vertebra to the overall Weberian structure in catfishes. While zebrafish (and other Cypriniformes) do not incorporate vertebra 5 into the Weberian apparatus to the extent of siluriform species, taken broadly, it is clear that vertebra 5 should, at a minimum, be considered a transitional vertebra between classically defined Weberian vertebra and posterior rib‐bearing vertebrae, and the modifications suggest that this vertebral level is responsive to the Weberian developmental module.

### 4.2 Possible functional roles of the V5 intercostal ligament and updated model

There are several anatomical features that both distinguish ICL1 from more posterior intercostal ligaments and point strongly to a unique role related to the function of the Weberian apparatus. First, the shape differences between ICL1 and more posterior ICLs are distinct, with ICL1 being more cylindrical, while posterior ICLs are broader and flatter (Figure 2). Second, ICL1 has much different orientation, being nearly vertical in the dorsal–ventral axis, while posterior ICLs are predominantly flattened along the horizontal axis. Third, while posterior ICLs attach between ribs broadly onto small flanges in the ribs, ICL1 attaches in defined, deep depressions in parapophysis 4 and rib 5 (Figure 2b–e), likely allowing for more directed and focused action. These differences clearly point to a shift in function for ICL1. When paired with the slight medial direction at its parapophysis 4 attachment relative to its origin, this angle puts ICL1 in alignment with the os suspensorium, making it likely to respond to the movement of the os suspensorium resulting from swim bladder pulsation. Given that parapophysis 4 forms a synchondrosis with centrum 4 (Bird & Mabee, 2003), there is some theoretical rotation possible at that articulation. It is plausible that ICL1 is acting to limit unnecessary rotation of parapophysis 4, potentially in multiple planes, to prevent vibrational signal loss due to spurious movement of the surrounding skeletal structures of the Weberian apparatus, thereby helping to direct movement along the ossicle chain and increase sensitivity with minimal signal loss.

### 4.3 Conclusions and future directions

This and other recent studies (Bird, Abels, & Richardson, 2020; Bird, Richardson, & Abels, 2020) underscore the need for increased focus on the ligamentous components of the Weberian apparatus. For a truly comprehensive understanding of the Weberian apparatus and how it functions, more integrative and holistic approaches are needed that include all regions (ear, vertebrae, swim bladder) and all ligaments. We outline several areas of needed future research below with a focus on the ligaments.

#### 4.3.1 Comparative

In order to create realistic and testable functional hypotheses regarding how each of the Weberian ligaments, including ICL1, function independently and collectively, a detailed examination of ligament variability is needed. Given the lack of ICL1 in the descriptions of the Weberian apparatus in cypriniform fishes, a reexamination of vertebrae 4–5 is needed to properly assess both the presence of this ligament across species, as well as the structural connectivity of ICL1 with rib 5 and parapophysis 4. Primary focus should start with species possessing the “Open” Weberian morphology (Bird, Abels, & Richardson, 2020), which are most likely to have a structural similarity to the zebrafish. Surveys should then extend to those with anterior plates, such as gyrinocheilids, catostomids, and some botids. A much broader survey of the fate of ICL1 could then be undertaken on encapsulated species (cobitids, nemacheilids, balatorids, etc), as well as other otophysan clades such as Characiformes to determine the overall incorporation of ICL1 into the Weberian apparatus in disparate species.

The precise role of the os suspensorium in the function of the Weberian apparatus has remained elusive. In the zebrafish, the os suspensorium is deeply embedded within the tissue surrounding the swim bladder and is connected to the tripus and swim bladder tunica via the triple ligament (Figure 5; Bird, Richardson, & Abels, 2020). In addition, parapophysis 4, which bears the os suspensorium, is connected to the articular process of the tripus via the suspensor ligament. Still less is known about the possible role of rib 4. Given that parapophysis 4 articulates with centrum 4 via synchondrosis, this joint is potentially mobile. One possible role of rib 4 would be to provide balance to the entire vertebra 4 structure, and the added mass could provide inertial resistance to excessive motion of the os suspensorium; however, this and other hypotheses regarding the functions of the os suspensorium and rib 4 require empirical testing.

#### 4.3.2 Molecular/genetic

Comprehensive ligament molecular marker characterization and fiber composition studies are needed. Known fibers in the various ligaments include collagens, elastin, and others. Immunohistochemical and in situ hybridization analyses in these ligaments would confirm the presence of specific fiber types. In addition, ratios of the fiber content in the various ligaments would allow for testable hypotheses regarding tensile strength, which can then be related to overall function.

Regarding the patterning of the Weberian apparatus as a whole, there have been some limited studies in the context of somite patterning (Akama et al., 2020), sclerotome development (Leyhr, Sanchez, et al., 2023; Waldmann et al., 2021), and thyroid hormone regulation (Kapitanova & Shkil, 2014; Keer et al., 2019). However, while classic mutant analyses have been available for years for the inner ear (Malicki et al., 1996; Whitfield et al., 1996) and swim bladder (Winata et al., 2009), the genetic basis of Weberian apparatus development at the level of specific ossicles remains understudied. Additional genetic studies of the Weberian apparatus development are required to understand how the ossicles are shaped and the different connected ligaments obtain their molecular identities.

## AUTHOR CONTRIBUTIONS

J.L. and T.H. contributed to the conceptualization of the study. All authors participated in data collection, data analysis, interpretation, and drafting of the original manuscript.

## CONFLICT OF INTEREST STATEMENT

The authors declare that they have no conflict of interest to report.

## Data Availability

The data that support the findings of this study are openly available in European Synchrotron Radiation Facility Data Collection at https://data.esrf.fr/doi/10.15151/ESRF‐DC‐1057386678 (Leyhr, Tafforeau, et al., 2023).
